# Laniakea@ReCaS: exploring the potential of customisable Galaxy on-demand instances as a cloud-based service

**DOI:** 10.1186/s12859-021-04401-3

**Published:** 2021-11-08

**Authors:** Marco Antonio Tangaro, Pietro Mandreoli, Matteo Chiara, Giacinto Donvito, Marica Antonacci, Antonio Parisi, Angelica Bianco, Angelo Romano, Daniela Manila Bianchi, Davide Cangelosi, Paolo Uva, Ivan Molineris, Vladimir Nosi, Raffaele A. Calogero, Luca Alessandri, Elena Pedrini, Marina Mordenti, Emanuele Bonetti, Luca Sangiorgi, Graziano Pesole, Federico Zambelli

**Affiliations:** 1 Institute of Biomembranes, Bioenergetics and Molecular Biotechnologies, National Research Council (CNR), Via Giovanni Amendola 122/O, 70126 Bari, Italy; 2 National Institute for Nuclear Physics (INFN), Section of Bari, Via Orabona 4, 70126 Bari, Italy; 3 Department of Biosciences, University of Milan, Via Celoria 26, 20133 Milano, Italy; 4 Istituto Zooprofilattico Sperimentale Della Puglia e Della Basilicata, Via Manfredonia 20, 71121 Foggia, Italy; 5 National Reference Laboratory for Coagulase-Positive Staphylococci Including Staphylococcus Aureus, Istituto Zooprofilattico Sperimentale del Piemonte, Liguria e Valle d’Aosta, Via Bologna 148, 10154 Turin, Italy; 6 Clinical Bioinformatics Unit, Scientific Direction, IRCCS Istituto Giannina Gaslini, Via Gerolamo Gaslini 5, 16147 Genova, Italy; 7 Italian Institute of Technology, Via Morego 30, 16163 Genova, Italy; 8 Department of Life Science and System Biology, University of Turin, Via Accademia Albertina, 13-1023 Turin, Italy; 9 Department of Computer Science, University of Turin, Via Pessinetto 12, 10049 Turin, Italy; 10 Department of Molecular Biotechnology and Health Sciences, Via Nizza 52, 10126 Turin, Italy; 11 Department of Rare Skeletal Disorders, IRCCS Istituto Ortopedico Rizzoli, Via di Barbiano 1/10, 40136 Bologna, Italy; 12 Department of Biosciences, Biotechnologies and Biopharmaceutics, University of Bari, Via Orabona 4, 70126 Bari, Italy; 13 Present Address: Department of Experimental Oncology, European Institute of Oncology, Via Adamello 16, 20139 Milan, Italy

**Keywords:** Galaxy, Cloud computing, Bioinformatics, Laniakea, Software environment, NGS, ELIXIR, Covid-19, SARS-Cov-2

## Abstract

**Background:**

Improving the availability and usability of data and analytical tools is a critical precondition for further advancing modern biological and biomedical research. For instance, one of the many ramifications of the COVID-19 global pandemic has been to make even more evident the importance of having bioinformatics tools and data readily actionable by researchers through convenient access points and supported by adequate IT infrastructures. One of the most successful efforts in improving the availability and usability of bioinformatics tools and data is represented by the Galaxy workflow manager and its thriving community. In 2020 we introduced Laniakea, a software platform conceived to streamline the configuration and deployment of “on-demand” Galaxy instances over the cloud. By facilitating the set-up and configuration of Galaxy web servers, Laniakea provides researchers with a powerful and highly customisable platform for executing complex bioinformatics analyses. The system can be accessed through a dedicated and user-friendly web interface that allows the Galaxy web server’s initial configuration and deployment.

**Results:**

“Laniakea@ReCaS”, the first instance of a Laniakea-based service, is managed by ELIXIR-IT and was officially launched in February 2020, after about one year of development and testing that involved several users. Researchers can request access to Laniakea@ReCaS through an open-ended call for use-cases. Ten project proposals have been accepted since then, totalling 18 Galaxy on-demand virtual servers that employ ~ 100 CPUs, ~ 250 GB of RAM and ~ 5 TB of storage and serve several different communities and purposes. Herein, we present eight use cases demonstrating the versatility of the platform.

**Conclusions:**

During this first year of activity, the Laniakea-based service emerged as a flexible platform that facilitated the rapid development of bioinformatics tools, the efficient delivery of training activities, and the provision of public bioinformatics services in different settings, including food safety and clinical research. Laniakea@ReCaS provides a proof of concept of how enabling access to appropriate, reliable IT resources and ready-to-use bioinformatics tools can considerably streamline researchers’ work.

## Background

The availability and accessibility of analytical software tools, standardised workflows and IT infrastructures are among the most critical enablers of data-driven science [1]. Life sciences, in particular, have assisted in recent years to unprecedented growth in the volumes, variety and complexity of data produced due to the development of modern high-throughput technologies [2]. A parallel increase in the numerosity and complexity of the bioinformatics software tools required to analyse the data was also observed due to the need to manage different data formats, associated metadata descriptors and the interoperability of upstream and downstream tools. Furthermore, bioinformatics software is frequently made available as source code, often with multiple and concatenated dependencies. Advanced IT skills are usually necessary for the proper installation and configuration of bioinformatics tools and their usage, which usually requires a good knowledge of the *nix command-line shell. A factor that still represents a relevant limitation for a large portion of the potential user base. Similar considerations can be applied to bioinformatics workflows, which are an arrangement of software components that can be heterogeneous in their versioning, dependencies and environment requirements, limiting their portability, integration and, ultimately, making it challenging to ensure an adequate level of analytical reproducibility [3]. The bioinformatics community in recent years has devoted considerable efforts to mitigate those barriers, for example, with the development of workflow managers (e.g., Taverna [4], Chipster [5], Galaxy [6]) as well as tools and workflow registries and repositories (e.g., bio.tools [7], myExperiment [8], Workflowhub [9]).

However, access to data and tools may still not be enough when those cannot be supported by adequate IT infrastructure. An ever-growing number of initiatives offer life sciences researchers access to cloud [10] and HPC resources [11–13]. Nevertheless, their use can introduce additional layers of complexity and require at least basic knowledge of the front-end used for the submission of jobs and the underlying infrastructure (e.g., number of cores per computing node, RAM available to each node, etc.), even worse, different infrastructures may adopt different software and hardware layers.

In this context, the emergence of the COVID-19 pandemic [14, 15] is just the most recent example of how prompt, straightforward, efficient and structured access to bioinformatics data, tools and workflows supported by suitable IT infrastructures is critical for researchers and healthcare professionals [16, 17]. The need to quickly process large amounts of data and make results readily available highlighted, once more, how advanced platforms for the integrated management of bioinformatic analytical pipelines, as Galaxy, are highly efficient in providing adequate analytical capacity [18].

Galaxy is currently the most popular workflow manager for bioinformatics, supported by a thriving community of users, developers, and researchers that have evolved over the years this platform into a complete ecosystem offering, e.g., software repositories, events, training, support [6]. The Galaxy front-end organises and exposes analytical tools and workflows through an intuitive and easy-to-use graphical user interface that makes analytical reproducibility the core of its design and implementation philosophy [3]. Hundreds of bioinformatics tools have been already made compatible with Galaxy and can be easily deployed by the administrators of any Galaxy instance through the Galaxy ToolShed [19]. Also, Galaxy supports several resource managers [20], which can be used by the platform as a proxy to access local or remote cloud [21] and HPC [22] infrastructures. The UseGalaxy [23] public servers [6], which represent the main Galaxy instances managed directly by the Galaxy Community, offer academic users a fair amount of free computational resources that are generally enough for basic analytical needs on reasonably sized datasets. Further evidence of the quality and value of this ecosystem is provided by its spilling over the bioinformatics domain boundaries, as Galaxy is increasingly being adopted by other scientific domains [6].

However, there are several scenarios and use cases with requirements that public servers cannot reasonably be expected to meet. Including, for example, intermediate to large datasets (> 250 GB including intermediates according to the official Galaxy documentation [24]) and computational loads, high-level requirements for data security, installation of custom tools and reference data, prioritisation of particular job types, access to the underlying file system and OS for development of tools or Galaxy itself and complex training activities, e.g., involving many users or requiring custom tools. In all those cases, administrative control over the Galaxy instance would be helpful or altogether necessary, and some of them may also require access to the underlying software or even hardware frameworks. This level of control can be achieved by installing Galaxy locally, a course of action that requires time, expertise, and access to an adequate IT infrastructure that can be costly both to set up and maintain. A solution to this issue is represented by the increasing maturity and availability of cloud technologies [25] that offer the possibility of providing virtual hardware and software platforms as a service.

We recently developed Laniakea [26], a Galaxy “on-demand” software platform based on cloud technologies specific for scientific applications [25]. In brief, Laniakea allows its users to deploy a completely customisable instance of Galaxy in the cloud. That is, a user of Laniakea can become the owner and administrator of one or more production-grade Galaxy instances in a matter of minutes. The configuration of a Galaxy instance, both at the software and virtual hardware layers, is performed through a user-friendly web front-end. This interface guides the user through a small number of steps to deploy Galaxy instances ready to be used intensively by many users. Among other features, Laniakea provides the possibility of deploying Galaxy instances, which we call “flavours”, already tailored for specific scientific tasks, allows to bind Galaxy instances to a virtual computer cluster and support secure storage encryption for applications with high-level data security requirements, e.g. analysis of sensitive human genetic data.

Herein we present the results of the first year of activity of Laniakea@ReCaS, the first Laniakea-based Galaxy on-demand service that is provided and managed by ELIXIR-IT [27], the Italian Node of ELIXIR [28]. In the next section, we provide a brief overview of the service architecture. Then, we present eight use cases from several Italian institutions that used the service, and finally, we move on to our conclusions and future perspectives in the last section.

## Implementation

### The ELIXIR-Italy Laniakea@ReCaS open call

ELIXIR-IT and ReCaS-Bari [29] launched the Laniakea@ReCaS service and the associated open-ended call on February 2020 [30], offering cloud resources for the creation of production-grade Galaxy instances to accelerate the development of novel bioinformatics tools and services, and to facilitate large scale analyses of data produced in the context of different scientific projects.

Applications in the form of a short proposal can be submitted through a simple web form [31]. Applicants must provide a brief description of their project, including an estimate of the required computational resources (virtual compute cores, RAM and storage), the expected number of users for their Galaxy instance(s), and the estimated duration of the project. Projects are evaluated by a scientific and technical evaluation board appointed by ELIXIR-IT. Approved projects can access a package of Cloud resources dependent on the requirements of the application as follows:

- up to 32 CPUs, (minimum of 8 CPUs);
- up to 64 GBs of RAM, (minimum of 16 GB);
- up to 2 TBs of storage, (minimum of 500 GB).

Resources are allocated with a “first come, first served” policy until the total available resource budget is assigned. Once allocated, resources can be used to create one or more instances of Galaxy depending on the specific needs of each user.

### Laniakea and Laniakea@ReCaS

**Fig. 1:**
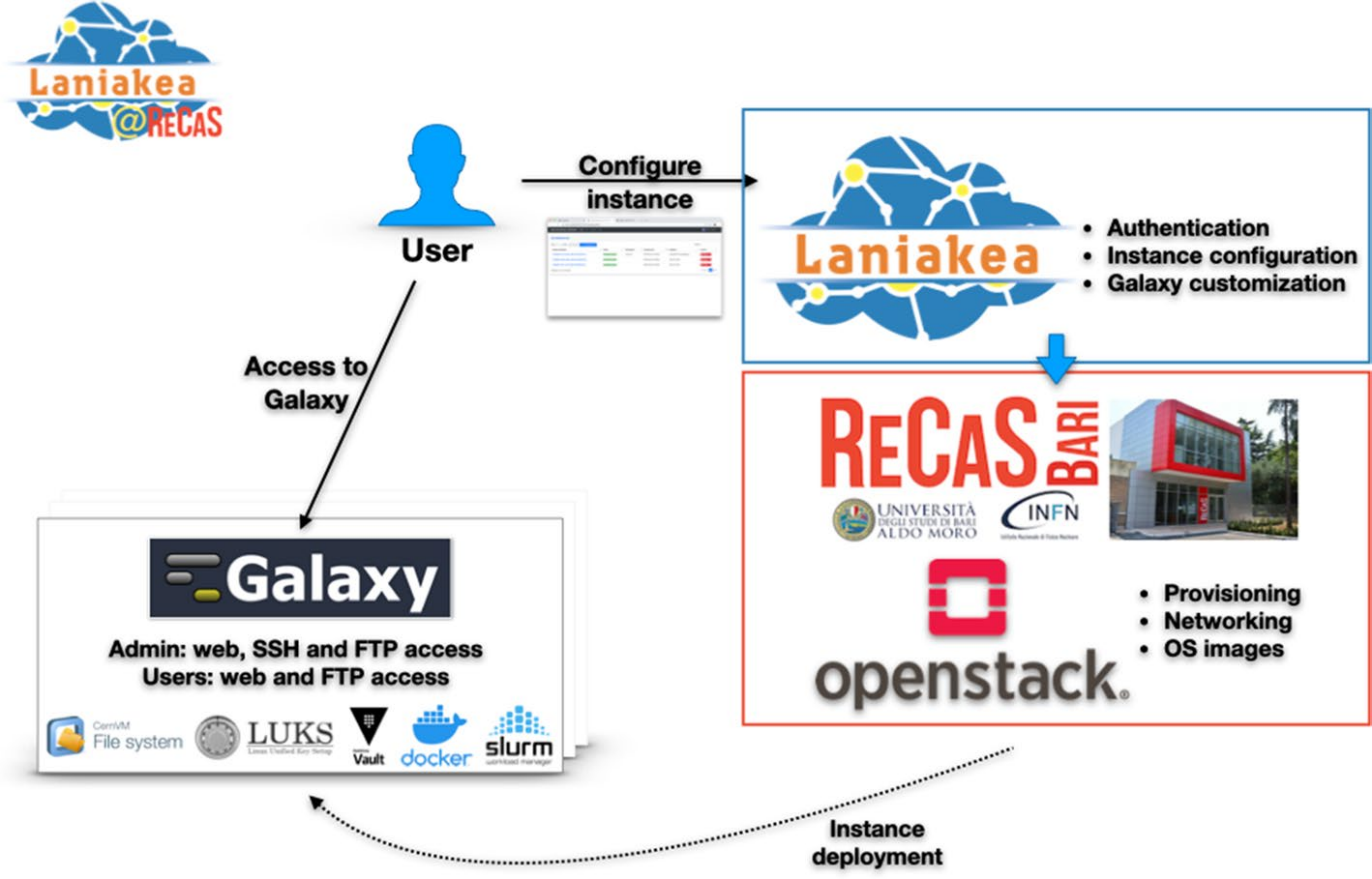
Laniakea@ReCaS architecture. Laniakea is installed on a dedicated OpenStack tenant on the ReCaS datacenter Cloud. Laniakea provides the Authentication and Authorization system and the dashboard that allows authorized users to configure the virtual hardware and software setup of their Galaxy instances. Once the user is satisfied with the configuration, Laniakea deploys virtual machines on the ReCaS Cloud, then installs and configures Galaxy, tools, and reference data, according to the user’s preferences

Laniakea@ReCaS (Fig. 1) leans on the Laniakea software platform [26]. In brief, Laniakea provides the virtual hardware and software orchestration services and their integration with the Authentication and Authorisation Infrastructure (AAI), automating the deployment, configuration, customisation and monitoring of the virtual Galaxy servers. The Laniakea web-based dashboard allows the customisation of virtual hardware and software layers associated with each Galaxy instance, including the Galaxy version (currently releases 17.05, 18.05 and 19.05 are supported, soon 20.05 and 21.05 will be added, too) and the administrator user's login credentials. Laniakea’s software stack and complete documentation are available at https://github.com/Laniakea-elixir-it and https://laniakea.readthedocs.io/en/latest, respectively.

Laniakea@ReCaS users can choose among several Galaxy flavours, that is, Galaxy instances customised with task-specific tools and workflows:

- Galaxy minimal: the standard Galaxy production environment;
- Galaxy COVID-19: COVID-19 analysis package [32];
- Galaxy Epigen: based on the Epigen project public server, provides a selection of tools for ChIP-Seq analysis [33];
- Galaxy RNA Workbench: more than 50 tools for RNA centric analysis [34];
- Galaxy CoVaCS: workflow for genotyping and variant annotation of whole genome/exome and target-gene sequencing data [35];
- Galaxy GDC Somatic Variant: porting of the Genomic Data Commons (GDC) pipeline [36] for the identification of somatic variants on whole exome/genome sequencing data.

Each Galaxy instance can be linked to a read-only volume, shared by all the Lanaiakea@ReCaS Galaxy instances and hosting reference data, while the private data volume associated with each instance can be encrypted to ensure high-level data protection. Finally, Laniakea@ReCaS supports the deployment of containerised [37] versions of Galaxy [34, 38] and the association of an instance with a virtual cluster to enable large-scale data analysis.

### The ReCaS-Bari datacenter

**Fig. 2:**
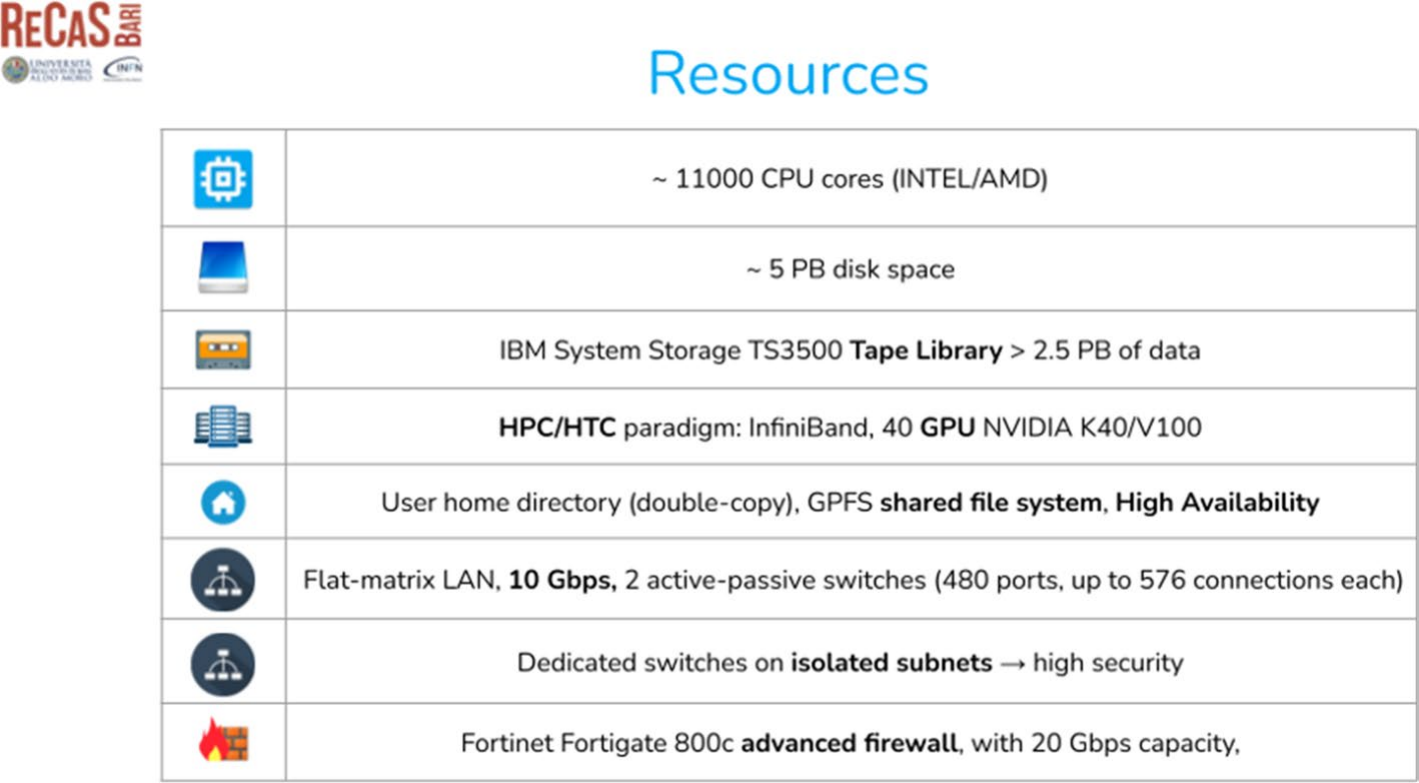
The total resource pool currently available at the ReCaS-Bari datacenter

The ReCaS-Bari data center [29, 39] (Fig. 2) is a key component of the IT infrastructure coordinated by ELIXIR-IT. Since 2015 ReCaS-Bari has hosted several services for ELIXIR-IT, including data storage and tools for data analysis. The ReCaS-Bari and INFN-Bari group have extensive experience in developing new scientific analysis services by using state of the art technologies and solutions [25, 40–45].

The Cloud Infrastructure as a Service implemented by the INFN-Bari, constitutes the backbone of Laniakea@ReCaS, and is used to provide the required levels of automation and scalability. To provide reliable, fast and secure access to the data, a dedicated storage system was developed specifically for the Laniakea@ReCaS service. Using a dynamic block storage solution this system provides high-availability of the data even in the case of loss of complete sets of disks. Moreover, an automatic and transparent encryption system was implemented to guarantee the safeguard of sensitive and/or personal data (i.e., clinical or human genetic data).

Funded by several European and national projects, the data center is gradually enhancing its resource pool and has plans that will make it able to provide up to 1000 CPU/core and 1Pbyte of storage for the Laniakea@ReCaS service in the next future. Furthermore, ReCaS will soon be able to allocate GPUs to speed-up the processing of applications that can benefit from GPU-computing and this service will be made available also to Laniakea@ReCaS users. The resources are made available via a private OpenStack installation that is able to accommodate the needed resources dynamically or through a Docker orchestration cluster (based on Mesos) when using bare-metal resources is critical for performance reasons.

## Results

**Fig. 3:**
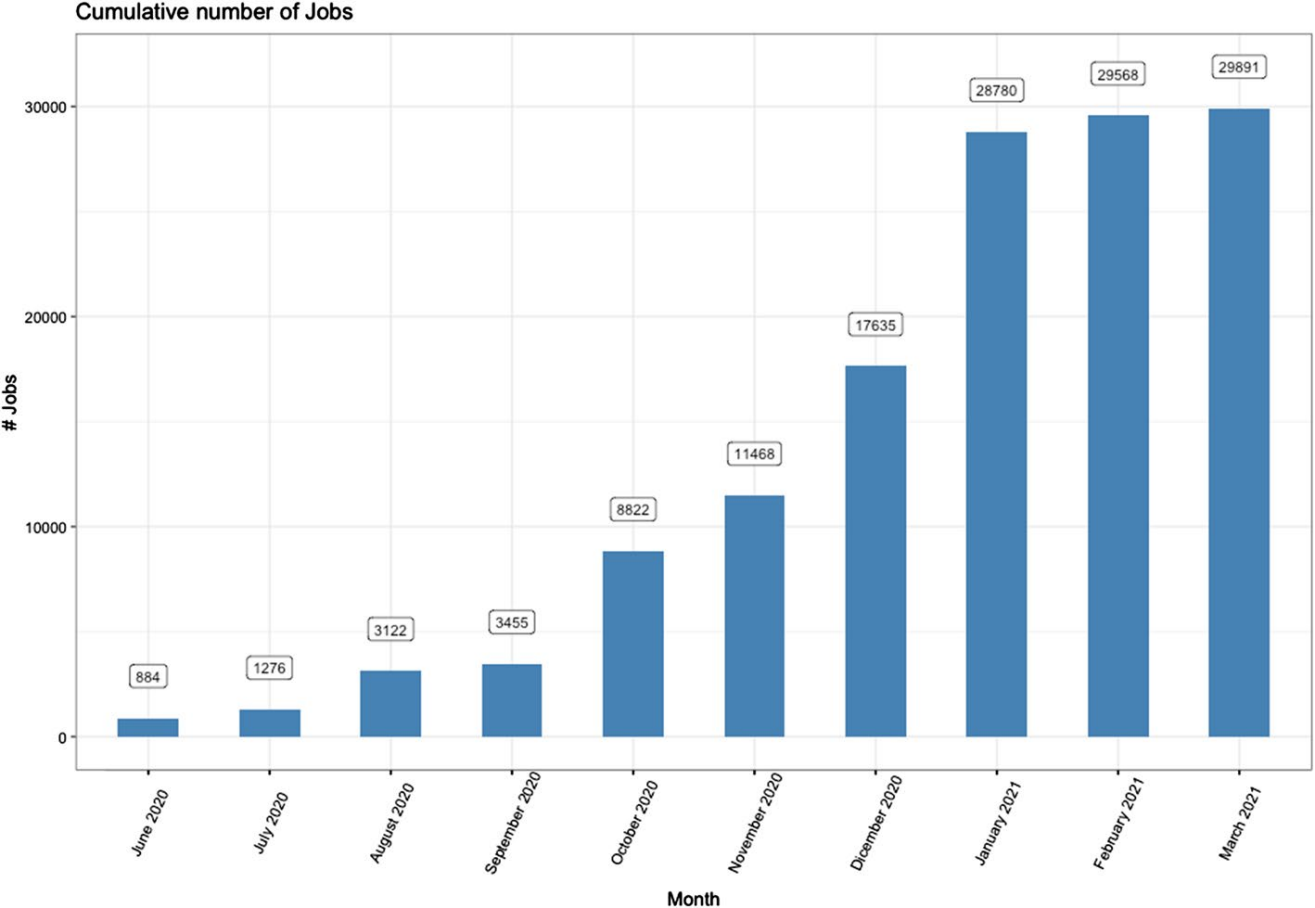
Cumulative number of jobs launched by all the Galaxy instances deployed on Laniakea@ReCaS up to March 2021

Since the opening of the open-ended Call in February 2020 [30], Laniakea@ReCaS has accepted ten project proposals for a total of 18 Galaxy instances operating on the ReCaS infrastructure that altogether launched almost 30 k jobs, as of March 2021 (Fig. 3).

In the following sections, the PIs of a selection of those projects will briefly describe their use case and how using Galaxy through the Laniakea@ReCaS service impacted their activity. The resources allocated to each use case and usage statistics are resumed in Table 1.

**Table 1:** Summary of the allocated resources and some usage statistics for the eight Laniakea@ReCaS use cases

	Use case 1 Vinyl UniMi	Use case 2 CorGAT UniMi	Use case 3 Genotyping bacterial species IZSPB*	Use case 4 S.I.R.I.O.IZS PLV*	Use case 5 L-PIPE-T IGG	Use case 6 Training UniTo	Use Case 7 rCASC UniTo**	Use case 8 Rare diseases IOR*
Quota: vCPUs Ram Storage	8 vCPUs 16 GB RAM 1 TB	8 vCPUs 16 GB RAM 200 GB	16 vCPUs 32 GB RAM 1 TB	16 vCPUs 32 GB RAM 1 TB	8 vCPUs 16 GB RAM 500 GB	32 vCPUs 64 GB RAM 1 TB	16 vCPUs 32 GB RAM 500 GB	32 vCPUs 64 GB RAM 2 TB
Users	23	17	5	11	20	30	3	4
Number of Galaxy instances	1	1	2	1 cluster (1 master node and 2 worker nodes)	1	2	1	2
Initial Galaxy flavour	Galaxy Minimal	Galaxy Minimal	Galaxy Minimal	Galaxy Minimal	Galaxy Minimal	Galaxy RNA Workbench	Galaxy Minimal	Galaxy CoVaCS
Jobs run (up to March 2021)	542	558	8159	989	5429	22,836	700	11,536

(*) This instance will be soon updated to increase compute and storage requirements

(**) This instance has been moved to a dedicated local server at University of Turin in February 2021

### Use case 1: variant prioritization

Applications of human genome sequencing technologies to healthcare and clinical practice are at the base of the development of novel, more accurate approaches to medical science, including, for example, personalized medicine [46]. The genome of a single individual typically carries millions of genetic variants with respect to the reference assembly of the human genome, all of which need to be carefully annotated and interpreted to identify those genetic variants potentially relevant from a clinical perspective [47]. *Variant prioritization* is a simple procedure commonly used in clinical studies to reduce the breadth of clinical genomics investigations. Briefly, a series of filters and criteria are established, based on the predicted functional effects of the variants, their overall prevalence in the human population and other relevant considerations, in order to retain only variants that are more likely to be associated with a pathological condition of interest [48]. While expert-designed guidelines for the interpretation and analysis of genetic variants in clinical settings are currently available, it is not uncommon for different operators to apply slightly different criteria and filters when performing variant prioritization, limiting the overall reproducibility of the results [49]. Additionally, the annotation of genetic variants depends largely on external databases and resources, which need to be regularly updated to obtain reproducible and accurate results [50].

To mitigate, at least in part, some of these issues and improve the reproducibility of variant prioritization in clinical studies, our research group from the University of Milan and the CNR Institute of Biomembranes, Bioenergetics and Molecular Biotechnologies (CNR-IBIOM) have recently developed VINYL (Variant prIoritizatioN bY survivaL analysis). This novel software suite integrates an innovative method for variant prioritization, along with a highly curated collection of databases and resources for the annotation of human genetic variants [51]. The main advantage of VINYL over other existing methods is that our tool can evaluate different scoring systems and metrics for the prioritization of genetic variants and provides a fully automated procedure to derive optimal criteria for the identification of genetic variants of potential clinical relevance. The system is flexible and allows the design of custom scoring schemes based on personalized functional annotations and can be adapted/optimized to different use cases and scenarios. Notably, extensive comparisons with equivalent state of the art methods demonstrated that VINYL could detect different types of genetic variants associated with pathological conditions and achieve higher levels of sensitivity and specificity than equivalent state of the art methods [51].

Notwithstanding the benefits outlined above, unfortunately, VINYL has some limitations, especially for what concerns its installation and maintenance. For example, the system comprises several distinct software modules used to perform different types of analyses: including annotation, scoring, and ranking of the candidate variants, along with methods for the graphical representation of the results. These tools are developed in different programming languages (mainly R and Perl) and require relevant IT skills to be correctly installed and configured. Additionally, VINYL depends on a large collection of publicly available databases and resources for the annotation of human genetic variants. These databases are large (globally more than 200 GB) and need to be updated regularly, making VINYL difficult to maintain and potentially impacting the reproducibility of the final results.

In the light of these considerations and to make the tool available to a broader user base, we decided to develop VINYL into a standalone workflow embedded in a Laniakea@ReCaS Galaxy instance. A substantial support/help was provided by the administrators of the service, both for the development of custom Galaxy wrappers and the installation of applications and software that were not already available into the main Galaxy toolshed. VINYL was converted into a fully functional standalone Galaxy instance in less than twenty days. All the required software and wrappers were also made available through Github to facilitate the installation and configuration of VINYL also on novel Galaxy instances [52]. A paper describing the method and the Galaxy instance was accepted for publication last December [51]. The VINYL Galaxy [53] service is officially up since the acceptance of the paper with no downtime. Currently, VINYL has 23 active users.

### Use case 2: functional annotation of SARS-CoV-2 genomic variants

Genomic surveillance, the ability to monitor the evolution of a pathogen in real-time and to identify and trace novel emerging mutations as they spread, currently represents one of the first lines of defence against the COVID-19 pandemic [17, 54]. At the time of writing (March 2021), more than 600 thousand complete or nearly complete genomic sequences of SARS-CoV-2, the etiological agent of COVID-19, are made publicly available through dedicated resources and databases [55]. Rapid and effective analysis of these data is of pivotal importance for recognizing relevant genomic mutations or SARS-CoV-2 lineages that might represent a concern for public health. Unfortunately, the majority of bioinformatics tools used for the functional annotation of SARS-CoV-2 genomic sequences were not devised explicitly for the analysis of viral genomes and presented some relevant limitations. For example, the specific mechanisms of transcription and post-translational processing of coronaviruses gene products [56] were not taken into account, resulting in the incorrect or incomplete annotation of several functional genomic sites, including conserved regulatory sequences [56] and non-coding secondary structure elements.

To overcome these limitations, our research group from the University of Milan and the CNR-IBIOM recently developed CorGAT—the Coronavirus Genome Analysis Tool, a novel, highly effective and user-friendly approach for the functional annotation of SARS-CoV-2 genomes [57]. Compared to equivalent methods, CorGAT incorporates a more comprehensive, manually curated collection of resources for the functional annotation of the genome of SARS-CoV-2 and provides additional layers of annotation missed by other tools for the functional annotation of SARS-CoV-2 genomic mutations [58]. These include variants associated with regulatory elements (transcription regulatory sequences, TRS), or to consensus cleavage sites [59] in the ORF1a and ORF1ab polyproteins and finally 161 variants in conserved secondary structure elements. By applying CorGAT to a collection of more than 70.000 complete SARS-CoV-2 genomes, we observed for the first time a possible functional shift or a loss of function of s2m, a conserved secondary structure element in the 3’ UTR which is shared by several coronaviruses and astroviruses.

To provide accurate and up-to-date annotations of SARS-CoV-2 genomic elements, CorGAT requires frequent updates to its “knowledgebase”. Consequently, locally installed versions of the tool might get out of date frequently. Additionally, while the tool is straightforward to use, CorGAT comes with a limited command-line interface, which might discourage users that are not familiar with *nix systems. To circumvent these limitations and make the usage of our tool more user-friendly and general, we decided to make CorGAT available also in the form of a dedicated Galaxy instance. A production-grade Galaxy instance of CorGAT was deployed through the Laniakea@ReCaS service in less than fifteen days. Similarly to VINYL, ad-hoc wrappers for the novel tools incorporated in CorGAT were developed with the help of the Laniakea development team. All the tools and wrappers are made available through a dedicated Github repository at [60].

A paper describing CorGAT was recently published [57]. The service is online [61], with no downtime since December 2020. Currently, we have 17 active users.

### Use case 3: rapid and comprehensive approach for genotyping bacterial species isolated from food

Foodborne pathogens, including bacteria, represent a relevant concern for public health and food safety. Food quality assessment is generally based on several well-established methods aimed at characterizing bacterial strains, such as serotyping [62, 63], antimicrobial susceptibility testing [64, 65], identification of relevant virulence factors and species identification by mass spectrometry [66]. Although these methods are commonly used for phenotypic characterization of pathogenic bacteria, they are extremely time-consuming. Furthermore, the above mentioned conventional approaches often fail to distinguish closely related isolates or to detect virulence/resistance features. This issue is primarily due to the limited genomic resolution of these molecular methods and the genetic features of the surveyed pathogens [67]. In this context, Whole Genome Sequencing (WGS) offers an invaluable resource for bacterial typing and genotyping. A plethora of tools and databases are available to accurately identify bacterial species and survey genetic elements related to drug susceptibility and virulence. Altogether, surveillance programs in food safety undoubtedly benefit from the availability of these tools since WGS allows for the early detection of known and emerging pathogens. Therefore, it is crucial to develop and use bioinformatics platforms that enable rapid and comprehensive genomic analyses. Our research group at the “Istituto Zooprofilattico Sperimentale della Puglia e Basilicata” (IZSPB) had the opportunity to use the Laniakea@ReCaS service to deploy a Galaxy instance dedicated to the identification and typing of several food pathogens (*i.e*. *Salmonella spp*, *Escherichia coli, Listeria monocytogenes, Staphylococcus spp*, *Enterobacteria*, *Bacillus cereus* group). We routinely employ public workflows available on the Galaxy instance to perform de novo assembly and annotation of bacterial genomes. For example, one advantage offered by the platform is the possibility of using the BTyper2 tool (version 2.3.2) [68], which is designed to perform a complete genotyping of strains belonging to the *B. cereus* group in one step, as described in Bianco and collaborators [69]. We also usually perform identification of antimicrobial resistance genes and virulence factors with ABRicate software [70], which includes several publicly available databases [71], NCBI AMRFinderPlus [72], CARD [73], ResFinder [74], and PlasmidFinder [75]. All analyses are launched and visualised in the web interface and subsequently downloaded; thus, data can be archived and maintained over time.

Based on our experience at IZSPB, the Laniakea@ReCaS service has successfully and rapidly allowed us to set up an efficient software framework for the reconstruction and functional annotation of genomes of bacteria identified in several food matrices. The platform made it possible to carry out rapid genetic characterizations of virulence or antimicrobial resistance genes in different microbial pathogens starting from the nucleotide sequencing data. Using the variety of available workflows and tools, we were able to rapidly classify, characterize, and assess the virulence potential of any isolate without necessarily possessing excellent bioinformatics skills or powerful computational resources. These considerations make Galaxy and the Laniakea@ReCaS service invaluable tools useful for performing complex analyses in different laboratories and, in prospective, even in clinical routine.

### Use case 4: Staphylococcus aureus Intensive server for Research In Omics data (S.I.R.I.O)

Whole Genomes Sequencing (WGS) can produce high-quality genome data in a short time and at a competitive cost. Due to this reason, this approach is becoming a common practice in microbiology with a significant impact on research, diagnostic and clinical microbiology. However, skills required to analyse and interpret the data are not common: while most laboratories have access to Next Generation Sequencing technologies, only a limited number have the infrastructure and specialised personnel required to analyse the data. Although many tools have now been developed for analysing NGS data, these tools often require bioinformatics skills that are not available to every laboratory. Even if commercial solutions are available, which offer a user-friendly interface and allow data analysis to staff without bioinformatics training, the cost of this software can be a real bottleneck for NGS use in routinely contexts. Moreover, since commercial platforms are based on proprietary software, there may be a gap regarding the transparency, reproducibility and sharing of the analysis. Due to these reasons, the Italian Reference Laboratory for Coagulase Positive *Staphylococcus* incl. *S. aureus* (IT-NRL CPS) based at the “Istituto Zooprofilattico Sperimentale del Piemonte, Liguria e Valle d'Aosta” (IZS PLV), decided to apply to the Laniakea@ReCaS open call to create a Galaxy instance called S.I.R.I.O. (*Staphylococcus aureus* Intensive server for Research In Omics data), also aiming at sharing the resulting Galaxy instance with the distributed network of equivalent laboratories in Italy (IIZZSS), that are involved in the activity of genomic characterization of bacterial strains. The S.I.R.I.O. Galaxy instance was implemented for harmonising and sharing the bioinformatics tools employed in the IIZZSS network and structured to offer the most widely used open-source bioinformatics tools for genomics in the microbiological field like FastQC [76], Trimmomatic [77], Unicycler [78], Check bacterial draft [79], SSPACE scaffolder [80], NCBI BLAST + integrated into Galaxy [81] and chewBBACA [82].

S.I.R.I.O. allows users with no specific bioinformatics skills or dedicated hardware to conduct computational intensive data analysis on bacterial genomes. Moreover, several workflows are shared with all the registered users, automating the analysis of raw sequencing data from the initial quality control of the sequences to the assembly and annotation of the genomes, and finally to perform phylogenetic inference in the event of microbial outbreaks. Thanks to the Laniakea@ReCaS service, the IT-NRL CPS laboratories network have the possibility to use high-throughput bioinformatic tools made accessible to "non-bioinformaticians'' personnel and perform comparative genomics analyses in a simple manner: all the laboratories now have the same capability of genomic characterization of foodborne bacteria. These advantages make Galaxy and the Laniakea@ReCaS service useful platforms for public pathogens-tracking laboratories like ours that have the necessity to perform genomic analysis routinary avoiding the need for a local physical infrastructure and highly specialized bioinformatics personnel. Currently, S.I.R.I.O. hosts 11 active users.

### Use case 5: L-PIPE-T: a PIPE-T-based service for the analysis of RT-qPCR expression data

Reverse transcription qPCR (RT-qPCR) is a standardised, sensitive, and fast method to quantify gene expression from qPCR experiments [83]. RT-qPCR experiments allow measuring the expression of several transcripts in parallel using high-density plates. Open-access software packages, tools, and web applications are currently available for the analysis of RT-qPCR data. However, the lack of a unified framework, non-trivial coding skills needed to use open-source solutions, and the absence of a simple framework for reusing, sharing, and communicating experimental procedures and results limit the reproducibility, transparency, and accessibility of the analyses. To fill those gaps, our research group at Istituto Giannina Gaslini has recently developed PIPE-T [83], the first Galaxy tool for parsing, filtering, normalising, imputing, and analysing RT-qPCR data. PIPE-T integrates the functionalities implemented in various R packages, such as HTqPCR, impute, and RankProd, in a simple, transparent, accessible, reproducible, and user-friendly environment. PIPE-T implements five distinct sequential procedures: file uploading and parsing, threshold cycle (Ct) filtering and categorization, data normalisation, transcript filtering and imputation, and differential expression analysis. PIPE-T is able to parse the main RT-qPCR file formats, including SDS, OpenArray, LightCycler, CFX, BioMark, and Plain and generate four tab-separated text files and three high-resolution images to assess quality control and identifying new potential biomarkers. We tested the ability of PIPE-T to analyze RT-qPCR data on two example datasets stored in the Gene Expression Omnibus repository [84] with accession identifiers GSE25552 and GSE43000. In both cases, our tool completed execution, returning the expected results.

In our experience, providing access to the tools via instances in public servers is a valuable strategy to access several tools, but in our case, this approach suffered from two limitations. On one hand, public instances did not allow us to install a novel tool like PIPE-T in a public instance. On the other hand, installing and maintaining a new public server would solve our problem, but it would require expensive computing resources, programming skills, and time. For these reasons, Laniakea@ReCaS was the most straightforward and fastest solution to create a customized Galaxy instance running PIPE-T. With less than 10 min of configuration, we launched a new working Laniakea@ReCaS Galaxy service running PIPE-T, referred to as L-PIPE-T. At the moment, L-PIPE-T hosts 20 active users who ran 538 analyses and stored 113 histories. L-PIPE-T was successfully used to identify new potential biomarkers of hepatic injury and inflammation in a murine model of glycogen storage disease type 1a [85].

### Use case 6: learning platform for undergraduate bioinformatics students

We are in charge of teaching bioinformatics to a broad audience, such as undergraduate students in biology at the University of Turin. With little setup effort by teachers, the Laniakea@ReCaS service made available to students the Galaxy web-based interface, allowing students to practice with bioinformatics concepts and algorithms, avoiding the steep learning curve needed to use UNIX shell, R or Python environments. The platform enabled biology-oriented students (without a specific computer science background) to run complex workflows, analyse real data and learn how to interpret the results in a learning-by-doing environment.

The cloud-based infrastructure of Laniakea@ReCaS proved to be an invaluable tool for teaching that, due to the COVID-19 pandemic, became the prevalent form of teaching academic courses in our country. Starting from publicly available databases or custom files shared with students through the Galaxy file-sharing system, students were able to follow laboratory lessons in teams from home, as well as practice alone when they preferred without the need to book computer rooms at the University.

Assessment tests were performed in the same environment, providing the students with real-world data to analyse, evaluating the knowledge acquired, and the competence developed and the skills mastered by students at the end of the course.

The analysis history logbook provided by Galaxy is particularly interesting; indeed, it allowed us to evaluate the progress of each student step by step, promptly identifying points that showed specific difficulties during lectures and exercises, and to check for cheating during exams.

To allow concurrent practical exams for more than 30 students effortlessly, we developed a custom procedure to replicate Galaxy virtual machine images (including user authentication data and shared files). The course contributed to the realisation of the objectives of the Biological Sciences Course, providing the students with basic knowledge in the field of bioinformatics. The Laniakea@ReCaS service was used by students and teachers to perform simple tasks (like aligning two protein sequences) as well as entire NGS pipelines as RNA-seq, ChIP-seq, variant-calling, including some downstream analysis like GO enrichment and KEGG pathway analysis.

### Use case 7: porting rCASC (reproducible classification analysis for single cells) to Galaxy: a complete analysis workflow facilitating single-cell RNAseq data analysis

Single-cell RNA-seq (scRNAseq) is a very powerful instrument to depict the overall cell complexity of healthy and disease tissues [86]. scRNAseq has today many different facets, spanning from full transcripts single-cell sequencing [87] to spatial transcriptomics [88] via droplet-based technology [89]. Different types of scRNAseq methods require dedicated data analysis workflows, which often are not user-friendly enough to be handled by life scientists with limited coding skills. rCASC [90–92] was developed at the University of Turin to provide a friendly environment to life scientists for the analysis of multiplatform scRNAseq, granting functional and computational reproducibility [93]. rCASC provides a complete set of analysis tools and pipelines allowing: i) conversion of raw data in count table, ii) cells’ quality control, iii) preprocessing, iv) normalisation, v) clustering, vi) cluster-specific markers detection, vii) biological knowledge extraction [92]. One of the peculiarities of rCASC is the possibility to evaluate clusters’ robustness via the cell stability score (CSS) [90]. The 88 tools and functions of the rCASC workflow are currently packaged as Docker containers, while input, output and tools are managed through R scripts. In this use case, we are at work making the whole workflow compatible with Galaxy leveraging the Laniakea@ReCaS service and are currently at one-third of the effort. For example, we have recently finished the porting in Galaxy of the new rCASC data mining instrument based on Sparsely Connected Autoencoders (SCA) [92]. This mining tool allows the identification of elusive players of cell clusters formation, such as transcription factors and miRNAs. CSS and SCA require the execution of multiple clustering jobs, making it difficult to perform such tasks onto conventional laptops. The Galaxy implementation of rCASC, which we are developing at Laniakea@ReCaS, offers at the same time a user-friendly environment and the possibility to customise Galaxy instances optimized for this specific analytical task and the dataset under analysis. Finally, due to the COVID-19 pandemic, all on-site courses we were running as part of our collaborations with EMBL and ELIXIR were cancelled. We are now finishing the preparation of a scRNAseq online course in collaboration with the training team of EMBL (Heidelberg) and, thanks to the rCASC implementation in Galaxy developed through Laniakea@ReCaS, we will be able to offer a practical training platform to the course participants.

### Use case 8: Rare diseases mutation detection

Rare diseases represent an emerging global public health priority. Thus far, 6000–7000 distinct Rare Diseases (RDs) have been recognised. Cumulatively RDs affect between 4–6% of the European population, ~ 70% of RDs are genetically inherited, and 69% have a pediatric-onset [94]. Even if therapies for RDs are often limited, reliable and rapid molecular diagnosis is necessary to avoid unnecessary tests and inadequate care. Genome/exome sequencing by NGS platforms provides a revolutionary diagnostic approach in this respect.

A key limitation in using NGS in clinical human genetics settings is the requirement of accurate and easy-to-use tools for the analyses of the large amount of data produced by high-throughput technologies, especially if we consider the potential lack of dedicated bioinformatics specialists. The aim of the case study by Istituto Ortopedico Rizzoli (IOR) was to develop a highly reproducible and reliable framework to facilitate the functional annotation and prioritization of genetic variants in patients affected by skeletal rare diseases. The genetic diagnosis of skeletal RDs is very complex: a limited number of causative mutations is currently known, and the discovery of novel potentially pathogenic mutations is limited due to both the small size of the cohorts of individuals affected by these pathologies and the lack of a specific and reliable estimate of background allele frequency distributions for the Italian population. Another specific aspect of our case study was the use of the Ion Torrent® sequencing technology. This NGS platform is associated with an excess of false-positive indel variant calls [95] and requires the application of dedicated tools and strategies for variants filtration. We customised the CoVaCS [35] Galaxy instance for “variant calling” provided by the Laniakea@ReCaS service to respond to those needs. Since CoVaCS is optimised to work primarily with data produced by Illumina platforms, the workflow was modified to take into account the error profile of the Ion Torrent technology, in particular with homopolymeric sequences. The following tools were added to the standards CoVaCS pipeline:

- *BamLeftAlign* (FreeBayes Package): a tool used to homogenize the positional distribution of insertions and deletions in the input using left realignment. Left realignment places all indels in homopolymer and microsatellite repeats in the same position, resulting in a more homogeneous and reproducible call of short indels at repetitive genomic sites. This method is inexpensive from a computational point of view and handles the most common classes of alignment inconsistency [96];
- *SnpSift Filter* (SnpSift Package): a tool for filtering VCF (Variant Calling Format) files using arbitrary expressions [97];
- *VCF to Tabular*: a custom-made tool for converting VCF files into tabular format (tab-delimited file).

Moreover, several databases and custom resources for the annotation of genetic variants were incorporated in a local copy of ANNOVAR that is used by CoVaCS, to provide functional annotation of genetic variants. These include gnomAD exome (version 211), a large collection of allele frequency estimates derived from the aggregation of exome sequencing data more than 125 thousand individuals [98], Intervar (v.20180118), a bioinformatics software for clinical interpretation of genetic variants according to the ACMG/AMP 2015 guideline [99], SPIDEX a computational model for the evaluation of the effect of genetic variants on splicing [100], and Hotspots. Hotspots has been developed ad hoc for the specific analysis of our case study. It facilitates recognising false positive calls by building, over time, a database of variant frequencies in the Italian population (data not yet published) which represents an essential support for both diagnostic and research purposes in the RDs scenario.

We reanalyzed 203 samples carrying a pathogenetic variant already identified and confirmed to validate our customised Galaxy instance. We focused our analysis on 91 samples with small insertion and deletion (INDELS) and multi-nucleotides variants (MNVs), since single nucleotide variants and splice-site single nucleotide variants did not require any modification of the CoVaCS pipeline. Importantly, the variant calling workflow based on our custom version of the CoVaCS pipeline, when compared to SeqNext (JSI medical systems GmbH, Kippenheim, Germany), an equivalent commercial software, displayed an overall higher sensitivity in the detection of all INDELS (CoVaCS 81.32%, SeqNext 78.02%), and in particular of single nucleotide INDELS (CoVaCS 88.52%, SeqNext 73.77%). While SeqNext showed higher levels of sensitivity in the detection of MNVs (CoVaCS 66.67%, SeqNext 86.67%).

In our experience, the Galaxy instance provided by the Laniakea@ReCaS service, integrated with the tools and datasets mentioned above, considerably sped up the data analysis process, making Galaxy easily usable by researchers and becoming a crucial support to conduct in-depth analyses of genetic variants in diagnostic and research contexts. By making sophisticated bioinformatics tools accessible also to researchers without a strong background in bioinformatics, like Galaxy, proved to be an essential tool for our use case. Since the lack of dedicated bioinformatics professionals and adequate computing resources is common to several public healthcare facilities in Italy, we anticipate that the tools and resources brought by the Laniakea@ReCaS project will be extremely useful for the Italian clinical research community. The availability of highly flexible and customisable bioinformatics pipelines offers a perfect solution for NGS applications in clinical settings, e.g. diagnostics, pharmacogenomics, personalized medicine, each one characterized by specific analytical needs.

## Conclusions

Laniakea@ReCaS provides researchers with a ready-to-use Galaxy environment backed by suitable computational and storage resources to handle their data analysis needs. As such, the service represents an example of a straightforward access channel to the computational resources provided by scientific cloud facilities and infrastructures, a channel that conveniently hides the complexity of the underlying software and hardware layers.

The maturity of the software layer and the reliability offered by the cloud environment has supported the daily work of several groups from different institutions across a range of applications spanning training activities, molecular diagnosis of rare diseases, food safety and bacterial characterisation, COVID-19 genomic functional annotation, and others.

One of the defining features of the service, as it emerges from most of the reported use cases, appears to be its customisability, that is the possibility for the user to freely and easily configure, modify, and manage the Galaxy environment. As such, this feature represents one of the most notable differences between a Galaxy on-demand service like Laniakea@ReCaS and a classic Galaxy public instance. Perhaps, the most interesting outcome made possible by this feature, one that we did not fully anticipate when Laniakea@ReCaS was launched, is that the service is being actively used as a platform to quickly develop and make available or more accessible to the community novel Galaxy based services as VINYL (use case 1), CorGAT (use case 2), Pipe-T (use case 5) and rCASC (use case 7).

Currently, Laniakea@ReCaS serves 18 Galaxy instances, for a total resource budget of 130 vCPUs, 250 GB of RAM and 5 TB of storage. This budget will be gradually increased, compatibly with the available funding, to support more use cases and meet future needs. We also aim at expanding and integrating the portfolio of available on-demand services for bioinformatics beyond Galaxy (e.g., with RStudio and Jupyter) by supporting them with Laniakea.

## Data Availability

Data sharing is not applicable to this article as no datasets were generated or analysed during the current study.

## Abbreviations

RT-qPCR: Reverse transcription quantitative real-time polymerase chain reaction
Ct: Threshold cycle
SDS: Sequence detection systems
EDS: Experiment detection systems
PIPE-T: Pipette
RDs: Rare diseases
VCF: Variant calling format
INDELS: Small insertion and deletion
MNVs: Multi-nucleotide variants
WGS: Whole genome sequencing
vCPU: Virtual Central Processing Unit
